# Health Economics at the Crossroads of Centuries – From the Past to the Future

**DOI:** 10.3389/fpubh.2016.00115

**Published:** 2016-06-09

**Authors:** Mihajlo (Michael) Jakovljevic, Seiritsu Ogura

**Affiliations:** ^1^Health Economics and Pharmacoeconomics, Faculty of Medical Sciences, University of Kragujevac, Kragujevac, Serbia; ^2^Department of Aging, Faculty of Economics, Hosei University, Tokyo, Japan

**Keywords:** health economics, history, future, health systems, health policy, twenty-first century, bibliography

## Abstract

Health economics, as an interdisciplinary science, has experienced exceptionally bold evolution through the past eight decades. Generations of committed scholars have built up huge body of knowledge and developed a set of methodological tools to assist health-care authorities with resource allocation process. Following its conception at the US National Bureau of Economic Research and Ivy League US Universities, this science has spread across the Globe. It has adapted to a myriad of local conditions and needs of the national health systems with diverse historical legacies, medical services provision, and financing patterns. Challenge of financial sustainability facing modern day health systems remains primarily attributable to population aging, prosperity diseases, large scale migrations, rapid urbanization, and technological innovation in medicine. Despite promising developments in developing countries with emerging BRICS markets on the lead, rising out-of-pocket health spending continues to threaten affordability of medical care. Universal health coverage extension will likely remain serious challenge even for some of the most advanced OECD nations. These complex circumstances create strong drivers for inevitable further development of health economics. We believe that this interdisciplinary health science shall leave long-lasting blue print to be visible for decades to come.

## Historical Roots of Health Economics

Health economics is the leading interdisciplinary science that bridges the gap between the theory of economics and practice of health care. It has experienced great development with earliest roots dating back for almost entire century. Its extensive diversification into various subdisciplines and areas of research endeavor is clearly visible today. Thus, it makes an uneasy task to track for its shy historical roots and preconditions for the birth of this science. Few would argue that actual cradle of this knowledge was academic tradition of the U.S.

Influential impact of the American institutions founded during the heights of Cold War rivalry extended far beyond their initially supposed outreach. One of the milestone events in the U.S. was establishment of Bureau of Medical Economics by American Medical Association (AMA) back in 1931. Shortly after the end of WWII, in 1945, the U.S. Army established the so-called RAND Project that grew in consecutive years to become RAND Corporation. Initially, its take was to apply mathematical Game Theory allowing for predictions of plausible scenarios in the geopolitical competition with the USSR. Many years after, applications of this same “rational choice” theoretical framework has found its civilian applications in areas such as the build-up of welfare state in liberal capitalism, social affairs, and ultimately economics of health care (1).

Some of the first pioneering works prior to WWII can be found in publications by Milton Friedman back in the 1930s (2, 3). Most commonly cited source responsible for name “baptism” of this science is Selma Muskin’s paper published 1958 entitled: “Towards the definition of health economics” (4) to be followed by her another famous paper “Health as an Investment” in 1962 (5). At that time, health was broadly regarded as rather consumptive branch of the economy. Mushkin’s analysis was probably the first understanding that health investment has long-term beneficial consequences for the community. Probably, single most famous and cited contribution laying grounds for this discipline was Kenneth Arrow’s paper “Uncertainty and the welfare economics of medical care” published in 1963 (6). These arguments were further extended by Michael Grossman who became known worldwide for authoring the Theory of Human Capital in 1972 (7). Interestingly, this method to assess indirect opportunity costs of lost productivity became standard in health economics. It continued to attract academic attention for many decades, and its creator even published two anniversary papers in recent years (8, 9). This same year was remembered for one more classical piece by Victor Fuchs entitled: Who Shall Live? (10). This contribution probably puts grounds for ethical consideration in health economics that are about to remain hot topics until today.

Particularly useful insight was provided by recent bibliographic study based on EconLit and Google Scholar indexing databases encompassing 40 years of health economics development from 1971 to 2011 published by Wagstaff and Culyer (11). It gives us thorough evidence on regional distribution of most relevant publications, top ranked academic institutions, and top cited researchers. Although discipline evidently accelerates since the 1960s, there is clear gap in favor of the U.S., which hosts almost 70% of top 100 researchers. It was distantly followed by Canada, UK, Australia, and afterward few continental European countries such as the Netherlands and Sweden. Top five most influential journals listed were Journal of Health Economics, Health Economics, Health Services Research, Pharmacoeconomics, and Journal of Human Resources. Top 10 most cited scientists globally according to multiple criteria (*h*-index, *I*^3^ factor, cumulative citation rate, etc.) were (in decreasing order of appearance) David M. Cutler, Jonathan Gruber, Paul Newhouse, Mark V. Pauly, W. Kip Viscusi, Janet M. Currie, Michael Grossman, Frank A. Sloan, Adam Wagstaff, and Eddy van Doorslaer. According to their *h*-index, the highest ranked institutions were Harvard University, World Bank, MIT, University of California at Berkeley, Chicago University, Pennsylvania University, Michigan University, and York University being the only European higher education establishment among the top ten.

Beginning from its very inception in the U.S. in between the 1940s and 1960s, health economics productivity continues to outpace European and that of major Asian economies, led by Japan (12). While the U.S. dominance in the output measures of health economics is still apparent, its monopoly is weakening for technical and substantive reasons. Technically, the high quality micro-data on individuals and households, essential for health economics that had been available only in the U.S., are now available in Europe and East Asian countries. Furthermore, even the latest quantitative tools in health economics find their way into these countries in no time through electronic journals and textbooks. However, most compelling reasons come from the huge differences between U.S. and the other countries, including Western Europe and Japan. U.S. is not a typical high-income country in the world; it has been almost the only country without universal coverage among OECD countries, many of its public health issues are unique, whose individualistic behaviors and values are often not shared by other countries. Countries with universal health insurance coverage often faced financial crisis much earlier due to population aging, and countries that followed simplistic text-book policy prescriptions often failed (13).

We should be aware that aforementioned bibliographic evidence published by World Bank, regardless of its undisputed methodological soundness, is some 5 years outdated. Since the late 1980s and the end of Cold War Era, health economics rapidly expanded worldwide (14). Leading engines of such development were academic centers of excellence whose establishment began in Western Europe (15) and Japan as early as in the 1970s (16). Furthermore, the process of mushrooming of scientific endeavor, funded research, establishment of under- and postgraduate curricula, and publication of textbook materials (17) in major world languages accelerated (18). Following traditional OECD economies (19, 20), new cores of fruitful efforts in the field occurred in major emerging markets such as the BRICS (21, 22) nations and Eastern Europe (22). Some of the prominent examples of such countries hosting some of the top 25 ranked centers of excellence were Taiwan (China), South Africa, China, and India (23). On the opposite side, clearly recognized geographical areas presenting almost serious lack of health economist capacities and locally published knowledge are most of the sub-Saharan Africa and Middle Eastern and Central Asian regions (24).

Large part of preconditions for such an extensive evolution were legislative changes across the Globe driven by the increasing costs of medical cares, slowing-down in economic growth, and the intensifying competition for the limited resources among the sectors of health care. Since the early 1990s, many governments, led by Australian (25) and Canadian (26) examples, adopted budget-impact and cost-effectiveness evidence as mandatory for new medical technologies approval and reimbursement. This wise decision effectively improved resource allocation strategies across the world, leading to better access and affordability of medical services to the general population (27). On the other side, it created an administrative pressure toward the manufacturers of pharmaceuticals and medical equipment. These, in turn, led to a great diversity of new job openings for professional industry-affiliated health economists (28). All these interconnected developments in the triangle of the national authorities, academia, and industry led to a great increase of capacities in terms of both knowledge and its applications.

Methodological mile stone essential to cost-effectiveness equation was reliable assessment of clinical efficiency of certain medical technology (29). Important step in this direction was revealing concept of evidence-based medicine (EBM) (30). It was applied straightforward in the UK *via* regular publication of Cochrane Systematic Reviews (31) and work of National Institute for Health and Care Excellence (NICE) (32). One step further in the same direction was development of health technology assessment (HTA) procedures (33). Establishment of national HTA agencies across the world throughout INAHTA-coordinated (34) efforts served as one of the long-term foundation stones, supporting the core place of health economics in health policy.

## The Changing World – New Challenges for the Global Health

Post WWII global development was characterized by several core landmarks of geopolitical evolution. Early decades were marked by demographic explosion, end of Colonial Era, 45 years of Cold War rivalry between the U.S. and USSR, rise of the Non-Aligned Movement (35), industrial revolution spreading from developed toward Third World economies (36), large scale migrations (37), environmental pollution (38), and ultimately global warming effect (39). Such a complex and dynamic socioeconomic evolution, both in free market capitalist and former socialist economies with some lag, led to technological advances and improved health outcomes with longevity on the lead (40). These major developments have accelerated hierarchical establishment and spreading of large national health systems across the world. The very idea of risk sharing and conception of early health insurance funds dates back to the nineteenth century Europe (41). After the WWII, the bulk of medical innovation moved from Europe to the U.S. Peculiarity is the fact that the first nation to effectively deliver universal health coverage for almost entire population including the poor, back in the 1930s, was USSR *via* its renowned Semashko system. Other countries adopted different strategies to provide universal health coverage – ranging from systems based on the state budget, through public insurance systems, to more market-oriented solutions with Bismarckian, Beveridge, and U.S. system being the most prominent examples. Great attainments in living standards, education, medical care, and public health policy led to bold advances in population health indicators. These were most visible in the U.S., Western European nations, and Japan at the time. They consisted of extended longevity, falling fertility rates, decreasing morbidity from infectious diseases, improved maternal health, and early childhood survival. With some gap, they were followed by the socialist countries. Low-income Southern Hemisphere nations of Africa, Latin America, and Asia remained significantly below these thresholds and needed decades to catch pace with the industrialized economies of the North (42).

Since the end of Cold War Era, observing the time horizon 1989–2016 World has become a much different place. During the 1990s and early 2000s, globalization processes continued to accelerate worldwide. This meant rise of the newly industrialized or the so-called “emerging” markets such as the BRICS (43) and Next Eleven nations (44), heavily dominated by the overachievement of People’s Republic of China (45). In health-care arena, it has reflected in so called transitional health-care reforms with the most typical pattern in Eastern Europe. These reforms tended to move focus of former socialist health systems from curative, hospital-based health care toward preventive-oriented, outpatient-centered health care. Health financing pattern tended to make relief to state-owned health insurance funds toward ever larger share of the out-of-pocket spending by the ordinary citizens (46).

Population aging as a global phenomenon (47, 48) became evident over the past three decades, with Japan on the lead (49, 50). Current UN forecasts indicate that China will be fastest aging large nation deep into the twenty-first century (51). Surprisingly, aging of nations ultimately reached some of the traditionally young nations such as the Arabic (52) ones of MENA region and Turkey (53). Such a huge demographic transition was virtually unpredictable for strategic thinkers of nineteenth century. Soon, it became obvious that this population change will be coupled with explosive increase in incidence and prevalence of non-communicable “prosperity diseases” (NCDs). Remarkable feature of core NCDs is their chronical clinical course characterized with growth in medical services demand, long lasting disability, work absenteeism, and premature mortality. The third major contributor to the skyrocketing costs of medical care provision was technological innovation in medicine. Diagnostic frontiers and therapeutic opportunities extended tremendously. Availability of cutting-edge technologies joined with improved affordability of ordinary “golden-standard” services leads to increased citizen demand, further threatening financial sustainability of contemporary systems. Cost containment efforts and polices soon became top policy priority not only in rich Western and Asian countries but elsewhere across the globe as well.

## Health Economics – Contribution to Modern Day Health Systems

It is broadly recognized that building of health care capacities has high, but long-term, return on investment in terms of population health and an overall societal productivity. Throughout the past half a century and more, the great legacy of academic health economics has created huge diversity of methodological tools to assist resource allocation processes. Some of the most commonly used were cost-effectiveness, cost-utility, cost-benefit, budget-impact, and resource utilization analysis. It continues to strive and create new approaches for the future such as improving management of scarce resources through managed and integrated care environment.

Health economics has established itself as a mature science long time ago. The landscape of its major subdisciplines remained essentially unchanged since the 1970s, although some areas were getting momentum more rapidly than others. This happened due to a myriad of local circumstances in the largest, most advanced health markets notably in Northern America, Western Europe, Far East of Asia, and Australia. To some extent, this focus of academic interest was driven by legislative framework and priorities of major research funding agencies (54–57). Another factor was pharmaceutical and medicinal device industry motive to invest heavily into those branches of health economics they regarded essential for the success of their market access and reimbursement strategies (58, 59). Last but not least, academia itself recognized the core weaknesses of national health systems and adapted responding to local needs (60). Thus, the major fields of scientific endeavor in health economics and its fruits were different in the U.S., Benelux, and Nordic regions of Europe and Japan. According to Wagstaff and Culyer based on EconLit-adopted JEL system, over the past 40 years, these disciplines were health and its value, efficiency and equity, determinants of health and ill-health, public health, health and the economy, health statistics and econometrics, demand for health and health care, health insurance, supply of health services, human resources, and markets in health care and economic evaluation.

Great legacy of academic health economics shall remain one of the prime examples of bold development of interdisciplinary health sciences. It has served governments, hospital sectors, and pharmaceutical and medicinal device industry across the world for more than half a century. This knowledge has facilitated wiser and more just resource allocation. It allowed to the national health systems worldwide to deliver more medical care, save more lives, extend human longevity, and improve the quality of life in patients suffering from chronic illness probably as much as core medical discoveries themselves. This contribution is less obvious compared to the innovation in clinical medicine. But far-reaching consequences of implementing methods of health economics in policy decisions have made medical services far more affordable and accessible to ordinary people than ever before (61, 62). Thus, a scientific discipline, originally stemming from the Game Theory, gave birth to several generations of gifted and devoted economists, physicians, and other expert profiles. All of them were building huge body of knowledge that continues to grow further. We witness complex evolution of global population health landscape across nations. Such a profound and long-term change creates a major challenge of financial sustainability of modern day health systems. Contemporary health policy elites believe that achievement of universal health coverage remains primary goal (63). We believe that these circumstances create strong long-term drivers for inevitable further development of the science of health economics. For many years, it has been supporting the build up of welfare societies in many nations (64). Whether health economics shall remain in the elite club of interdisciplinary health sciences exhibiting long-lasting impact to policy-making and strategic thinking will be seen in the upcoming decades.

## Author Contributions

MJ and SO contributed equally in study design, conception, and drafting of the manuscript. Both authors had essential contributions to the final appearance of this grand challenge article.

## Conflict of Interest Statement

The authors declare that the research was conducted in the absence of any commercial or financial relationships that could be construed as a potential conflict of interest.
